# Activated amino acid response pathway generates apatinib resistance by reprograming glutamine metabolism in non-small-cell lung cancer

**DOI:** 10.1038/s41419-022-05079-y

**Published:** 2022-07-21

**Authors:** Xiaoshu Zhou, Rui Zhou, Xinrui Rao, Jiaxin Hong, Qianwen Li, Xiaohua Jie, Jian Wang, Yingzhuo Xu, Kuikui Zhu, Zhenyu Li, Gang Wu

**Affiliations:** 1 Cancer Center, Union Hospital, Tongji Medical College, Huazhong University of Science and Technology, Wuhan, 430022 China; 2 Institute of Radiation Oncology, Union Hospital, Tongji Medical College, Huazhong University of Science and Technology, Wuhan, 430022 China

**Keywords:** Non-small-cell lung cancer, Cancer therapeutic resistance, Cancer therapeutic resistance, Cancer metabolism

## Abstract

The efficacy of apatinib has been confirmed in the treatment of solid tumors, including non-small-cell lung cancer (NSCLC). However, the direct functional mechanisms of tumor lethality mediated by apatinib and the precise mechanisms of drug resistance are largely unknown. In this study, we demonstrated that apatinib could reprogram glutamine metabolism in human NSCLC via a mechanism involved in amino acid metabolic imbalances. Apatinib repressed the expression of GLS1, the initial and rate-limiting enzyme of glutamine catabolism. However, the broken metabolic balance led to the activation of the amino acid response (AAR) pathway, known as the GCN2/eIF2α/ATF4 pathway. Moreover, activation of ATF4 was responsible for the induction of SLC1A5 and ASNS, which promoted the consumption and metabolization of glutamine. Interestingly, the combination of apatinib and ATF4 silencing abolished glutamine metabolism in NSCLC cells. Moreover, knockdown of ATF4 enhanced the antitumor effect of apatinib both in vitro and in vivo. In summary, this study showed that apatinib could reprogram glutamine metabolism through the activation of the AAR pathway in human NSCLC cells and indicated that targeting ATF4 is a potential therapeutic strategy for relieving apatinib resistance.

## Introduction

In recent years, reprogrammed energy metabolism was increasingly perceived as hallmark of cancer and associated with the tumorigenesis and progression of non-small-cell lung cancer (NSCLC) [1]. Chemotherapy, radiotherapy, and targeted therapy have all been found to alter tumor metabolism, and metabolic changes are often implicated in treatment resistance [2, 3]. Drugs targeting tumor blood vessels have been commonly used in NSCLC, although they have limited overall survival benefits for patients primarily because patients are susceptible to resistance to angiogenic therapy [4]. Anti-angiogenic drugs (AADs) have been found to enhance the depletion of oxygen and nutrients in tumor tissues and the formation of anoxic microenvironments, thus triggering metabolic changes that provide energy for tumor cells and promoting cancer progression. For instance, the classic anti-VEGF drug bevacizumab has been reported to promote glucose uptake and lactate production by upregulating the expression of hypoxic and glycolytic markers in glioblastoma [5]. Bevacizumab was also found to significantly impair mitochondrial function and activate glycolysis in colorectal cancer, and the glycolysis inhibitor 3-BrPA could relieve drug resistance to bevacizumab [6]. AADs could also facilitate lipid metabolism by promoting the uptake of free fatty acids, which stimulated the proliferation of colorectal cancer. The inhibition of fatty acid oxidation rate-limiting enzymes significantly sensitized the therapeutic efficacy of AADs [7].

Apatinib, a small tyrosine kinase inhibitor (TKI), selectively suppresses vascular endothelial growth factor receptor-2 (VEGFR-2) and other TKIs including c-Kit, Ret, and c-Src. Multiple studies have revealed that apatinib exerts antiangiogenic and antiproliferative effects in various solid tumors. The results of two multicenter phase III clinical trials indicated that apatinib significantly improves the survival of patients with advanced gastric cancers and hepatocellular carcinoma [8, 9]. Apatinib alone or in combination with chemotherapy/immunotherapy also exhibited ideal efficacy and controllable safety in NSCLC according to several clinical trials [10, 11]. In preclinical studies, apatinib was found to induce apoptosis and autophagy in osteosarcoma [12] and colorectal cancer [13], and the inhibition of autophagy was shown to promote the antitumor efficacy of apatinib. It was recently reported that apatinib could inhibit glycolysis by suppressing GLUT4 in ovarian cancer cells [14]. As an AAD, the effect of apatinib on metabolic balance remains unclear and deserves further exploration.

Amino acids (AAs) represent an essential nutrient for cell survival and growth. They are not only used as elementary unit for the synthesis of proteins, nucleotides but also play an important role in energy production and utilized as intermediate metabolism in mitochondria. Glutamine, which is a nonessential amino acid, plays a pivotal role in tumor cell metabolism by generating intermediate product drained into tricarboxylic acid (TCA) cycle and the nucleotide biosynthesis pathways. Additionally, recent studies have shown that glutamine mediates chromatin modification, regulates cell signaling and facilitates other amino acids transport [15]. In mitochondria, glutamine can be catabolized into glutamate and an ammonium ion by glutaminase (GLS). Although glutamine is synthesized de novo from glucose-derived carbons and ammonia, the amount of synthesized glutamine is insatiable for the requirement of several cancer cell, as it is known to be consumed in extensive amounts. Tumor cells become addicted to glutamine in their attempt to support exceptionally fast proliferation; thus, glutamine has been noted as “conditionally essential” [16]. However, the mechanisms underlying glutamine metabolism regulation by AADs have not been studied thoroughly.

Here, our study showed that apatinib treatment led to dramatic increases in glutamine metabolism in human NSCLC cells while glycolysis remained unchanged. First, the application of apatinib inhibited GLS1, the initial and rate-limiting enzyme of glutamine catabolism in cells, which activated the amino acid response (AAR) pathway (GCN2/eIF2α/ATF4). Overexpression of ATF4 elevated the expression of SLC1A5, a transporter of neutral AAs, especially glutamine, resulting in excessive intake of glutamine. ATF4 also upregulated ASNS, an enzyme that catalyzes the reaction of glutamine and aspartate to produce glutamate and asparagine. Mechanistically, the loss of ATF4 generated a global effect on cellular metabolism reprogramming, disrupted glutamine influx and catabolism, and induced autophagy and apoptosis in NSCLC cells. ATF4 knockout also decreased the progression of cancer in xenograft models of NSCLC. Because malignant cells present such strong reliance on AAR-mediated glutamine metabolism, this study suggests that ATF4 represents a promising therapeutic target for apatinib drug resistance. This mechanism of altering energy metabolism by apatinib may contribute to a better understanding of AAD resistance and cancer cell adaptations to nutritional restriction.

## Results

### In vitro experiment indicated Apatinib inhibited the proliferation of NSCLC cell lines

**Fig. 1:**
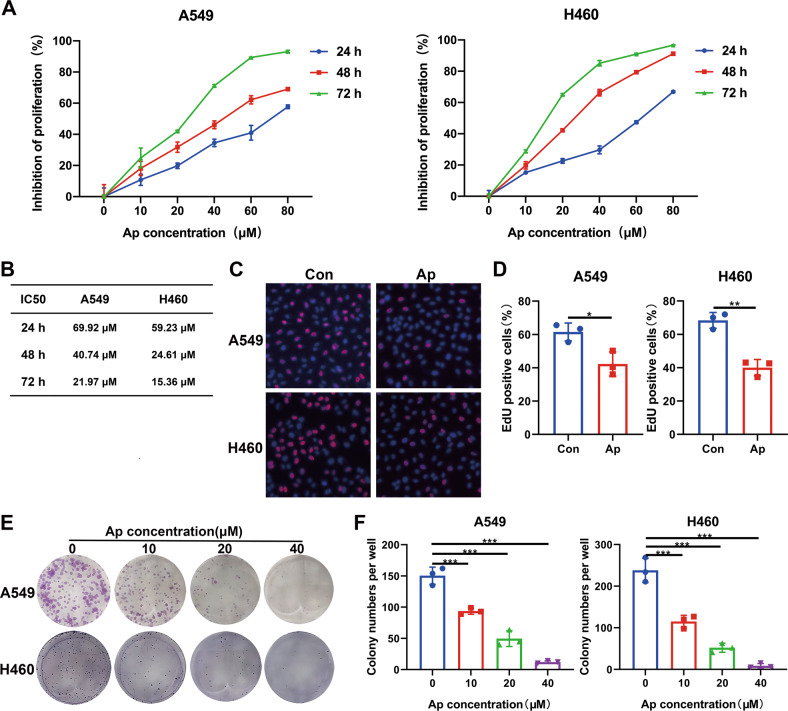
Apatinib inhibited the proliferation of NSCLC cells. **A** A549 and H460 cells were treated with apatinib at various concentrations (0, 5, 10, 20, 40 μM) for 24, 48, and 72 h. Viabilities of the cells were evaluated by CCK8 assays. **B** IC50 values of apatinib in A549 and H460 cells at 24, 48, and 72 h. **C**, **D** Representative images and statistical data of positive EdU staining (red) in NSCLC cells after treatment with 20 μM apatinib for 48 h. **E**, **F** The images and data statistics of the colony formation assay of A549 and H460 cells treated with apatinib. Data are presented as mean ± SEM from three independent experiments.

The antiproliferative effect of apatinib on A549 and H460 cell lines were evaluated by incubating cells with apatinib (0, 5, 10, 20, 40 μM) for 24, 48, or 72 h. The CCK-8 assays showed that the inhibition of apatinib on A549 and H460 cells occurred in a dose- and time-dependent manner (Fig. 1A). The IC50 of the A549 and H460 cell lines ranged from 15.36 to 69.92 μM at different concentrations and timepoints of apatinib treatment (Fig. 1B). The EdU detection assay was also used to label the cells undergoing DNA synthesis, which indicates active cell proliferation. The results showed that the percentage of red-labeled cells was decreased with the apatinib treatment (20 μM, 48 h) (Fig. 1C, D).

The colony formation assay further validated the anti-proliferative effects of apatinib on NSCLC A549 and H460 cells. After treatment with apatinib at various concentration for 48 h, the cells were cultured with fresh complete medium for another 7 days. As shown in Fig. 1E, F, the cell colonies decreased as the concentration of apatinib increased. All these results demonstrated that apatinib exerted an antiproliferative effect on NSCLC cells.

### Apatinib induced autophagy in NSCLC cells

To explore the antitumor effect of apatinib, we performed both apoptosis and cell cycle detection analyses. The A549 and H460 cells were treated with apatinib at various concentrations for 48 h. Our results demonstrated that apatinib is not able to cause apoptosis in human NSCLC cells at low concentrations (Fig. S1). Similarly, the cell lines were treated with apatinib for 48 h. The cell cycles were also analyzed by flow cytometry. Our results indicated that apatinib did not cause cell cycle redistribution at these concentrations (Fig. S2).

**Fig. 2:**
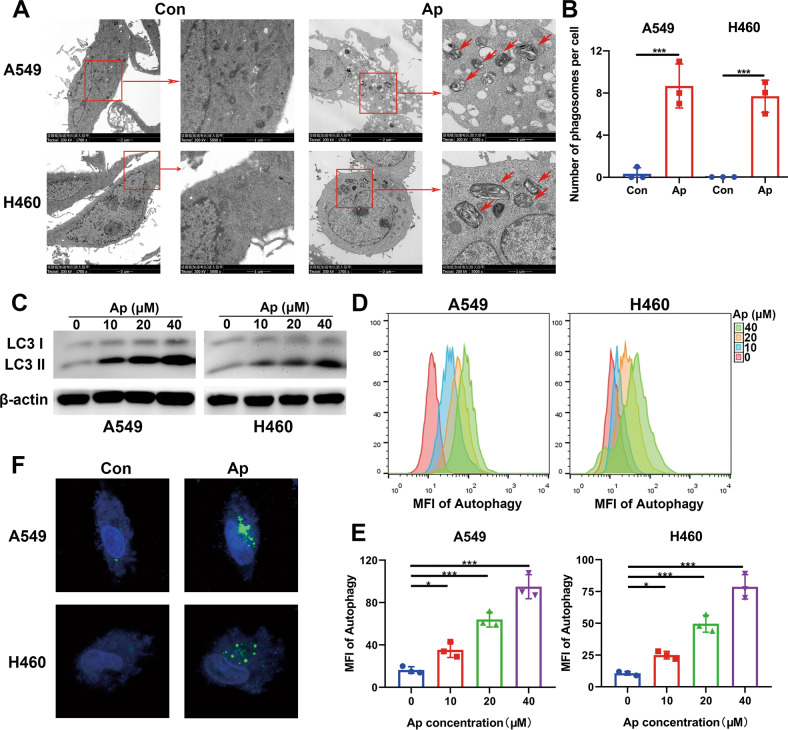
Apatinib induced autophagy in NSCLC cells. **A**, **B** Representative TEM images and analysis of the number of autophagosomes/autolysosomes of A549 and H460 cells with apatinib (20 μM, 48 h) or negative control incubation. Short red arrows: autophaosomes or autolysosomes. **C** LC3 I/II expression was detected by WB in A549 and H460 cells after treatment with incremental concentrations of apatinib for 48 h. **D**, **E** Cyto-ID detection assay measured the autophagy levels of A549 and H460 cells after treatment with incremental concentrations of apatinib for 48 h. **F** Representative images obtained by confocal microscopy of GFP-LC3 expression in A549 and H460 cells with apatinib (20 μM, 48 h) or negative control treatment. Data are presented as mean ± SEM from three independent experiments.

Meanwhile, we detected the microstructure of cells after apatinib treatment. Multiple autophagosomes or autolysosomes in the cytoplasm of apatinib-treated cells were observed by the transmission electron microscope (TEM) (Fig. 2A, B), which suggested that apatinib induced autophagy in NSCLC cells. The expression of LC3 II is positively proportional to the autophagy level. In general, LC3 I transforms into LC3 II when the autophagy level is increased. LC3 expression was detected by western blot (WB), and LC3 II expression was significantly increased with increasing apatinib dosage (Fig. 2C). Additionally, a Cyto-ID autophagy detection kit was used, and showed that the autophagy levels of A549 and H460 cell lines increased after apatinib treatment for 48 h (Fig. 2D, E). Finally, cell autophagy was further verified by transfection of cells with a GFP-labeled LC3 plasmid. GFP-LC3 fusion protein was normally dispersed and distributed in the cytoplasm. When autophagy occurred, the GFP-LC3 plasmid translocated and accumulated on the autophagosome membrane. As shown in Fig. 2F, vast green fluorescence foci appeared in the cytoplasm after treatment with 20 μM apatinib for 48 h.

### Apatinib activated alanine, aspartate, and glutamate metabolism in NSCLC cells

**Fig. 3:**
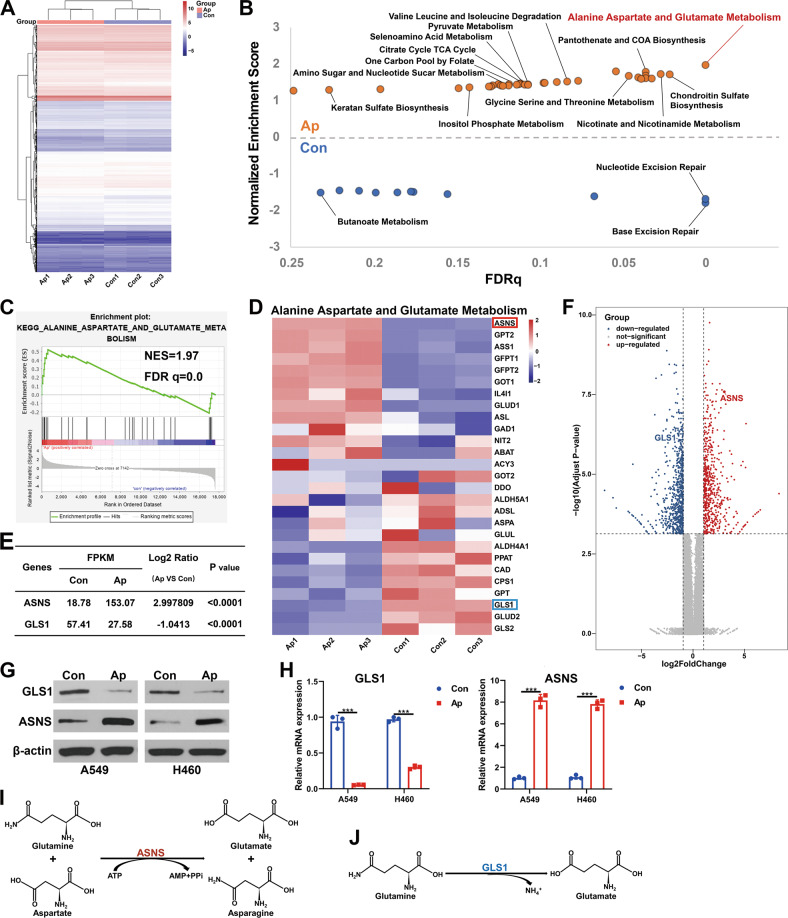
Apatinib activated alanine, aspartate and glutamate metabolism in NSCLC cells. **A** Hierarchical clustering (heatmap) of the differentially expressed genes (DEGs) of A459 cells with or without apatinib treatment (20 μM, 48 h). **B** Gene set enrichment analysis (GSEA) of metabolism-related genes in the A549 cells of both groups. The *X*-axis represents the FDR statistical value (FDRq), and the *Y*-axis represents the normalized enrichment score (NES). **C** GSEA of alanine, aspartate, and glutamate metabolism-related genes. NES, normalized enrichment score; FDRq, FDR statistical value. **D** Heatmap of the relative mRNA expression of alanine, aspartate, and glutamate metabolism-related genes. **E** mRNA expression of ASNS and GLS1 from the RNA-seq data. **F** Volcano plot of the DEGs. GLS1 and ASNS were marked up. The *X*-axis and the *Y*-axis represents log2 (fold change) and −log10 (adjusted *p* value), respectively. **G** Protein and **H** mRNA expression levels of GLS1 and ASNS detected by WB and qRT–PCR in A549 and H460 cells with apatinib (20 μM, 48 h) or negative control treatment. **I** Reaction catalyzed by ASNS. **J** Reaction catalyzed by GLS1. Data are presented as mean ± SEM from three independent experiments.

The RNA-Seq analysis were performed to explore the mechanism underlying the effects of apatinib on NSCLC cells. after apatinib treatment, the expression of 1334 genes showed a significant difference (|FoldChange| ≥ 2, *P* < 0.01), 578 genes were upregulated, and 756 were downregulated (Fig. 3A and Fig. S3A–C). A GO function analysis showed that there were differences in expression in cell biological processes, cell components, and subfunctions. In particular, we noticed that 544 genes were enriched in cell metabolism, among which the change in amino acid metabolism was the most significant (Fig. S3D, E). Through a KEGG pathway enrichment analysis, we also found that apatinib significantly affected several cell metabolic processes, especially the glutamine/glutamine metabolization-related pathway (Fig. S3F).

Further, gene set enrichment analysis revealed that multiple metabolic processes were activated after apatinib treatment (Fig. 3B and Fig. S3G, H). Among these pathways, the alanine, aspartate, and glutamate metabolism pathways were the most enriched (Fig. 3B, C; NES = 1.97, FDRq = 0.0). According to the genes enriched in this pathway (Fig. 3D), several key enzymes of glutamine metabolism were changed in apatinib-treated cells. Interestingly, we found that the expression of GLS1, a rate-limiting enzyme that catalyzes the decomposition of glutamine into glutamate (Fig. 3J), was significantly downregulated under the apatinib treatment. Another key enzyme, ASNS, which converts glutamine and aspartate to glutamate and asparagine in an ATP-dependent reaction (Fig. 3I), was upregulated (Fig. 3D–F). The results were confirmed by the WB analysis (Fig. 3G) and qRT–PCR analysis (Fig. 3H). Since GLS1 decreased while ASNS increased, we could not determine the exact content changes of intracellular glutamine and glutamate under apatinib treatment, which needs further exploration.

### Apatinib promoted the intake of glutamine by upregulating the expression of SLC1A5

**Fig. 4:**
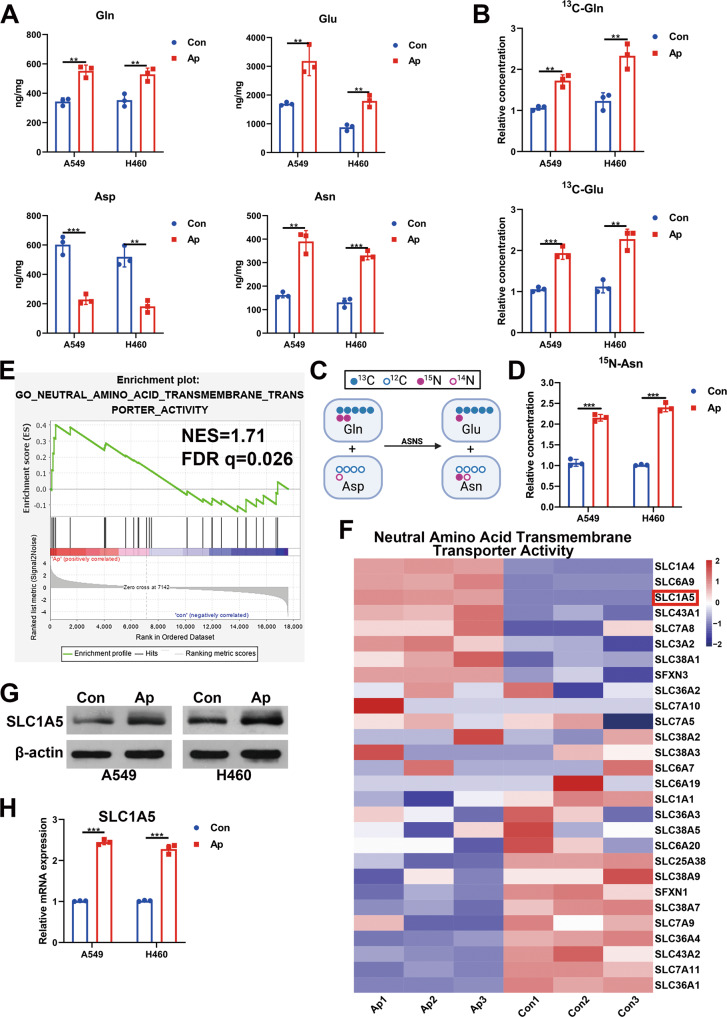
Apatinib promoted the intake of glutamine by upregulating the expression of SLC1A5. **A** Intracellular concentrations of glutamine, glutamate, asparagine, and aspartate in A459 and H460 cells incubated with apatinib or negative control (20 μM, 48 h) were detected by GC-FID. **B** LC–MS analysis of intracellular conversion of ^13^C-glutamine to ^13^C-glutamate in A459 and H460 cells incubated with apatinib (20 μM, 48 h) or negative control. **C** Schematic diagram of C and N metabolic flow in the reaction catalyzed by ASNS. **D** LC–MS analysis of the relative intracellular contents of ^15^N-labeled asparagine in control or apatinib-treated (20 μM, 48 h) A459/H460 cells. **E** GSEA of neutral amino acid transmembrane transporter activity-related genes. NES, normalized enrichment score; FDRq, FDR statistical value. **F** Heatmap of relative mRNA expression of neutral amino acid transmembrane transporter activity-related genes. **G** Protein and **H** mRNA levels of SLC1A5 detected by WB and qRT–PCR in A549 and H460 cells with apatinib (20 μM, 48 h) or negative control treatment. Data are presented as mean ± SEM from three independent experiments.

A GC-FID analysis was performed to detect the intracellular concentrations of twenty common amino acids in both A549 and H460 cells after apatinib treatment (Fig. 4A and Fig. S4). As shown in Fig. 4A, the content of aspartate was decreased and the content of asparagine was increased, which was consistent with the upregulation of ASNS. In order to definite whether the increase of asparagine was due to the slow proliferation or enhanced synthesis, we labeled cells with ^15^N-glutamine to measure the M + 1 asparagine flux and found the content of ^15^N-labeled asparagine was increased (Fig. 4D). Surprisingly, both glutamine and glutamate concentrations were increased significantly, which couldn’t be fully explained by the upregulation of ASNS. Glutamine is not the only source of glutamate; for example, α-KG can also be generated into glutamate under the catalysis of glutamate dehydrogenase [17]. Thus, to clarify the impact of apatinib on glutamine metabolism, we used exogenous ^13^C-labeled glutamine as the tracer and performed LC-MS detection. After the apatinib treatment, the concentration of ^13^C-glutamine and the concentration of ^13^C-glutamate increased (Fig. 4B). This finding suggested that there must be a compensatory glutamine supplementation pathway.

Hence, we performed a GSEA assay and found that neutral amino acid transmembrane transporter activity-related genes were enriched in the apatinib-treated group (NES = 1.71, FDRq = 0.026) (Fig. 4E). Among the numerous genes, we noticed that SLC1A5, the glutamine transporter, was upregulated (Fig. 4F). The increases in the protein and mRNA levels were further verified by WB analysis (Fig. 4G) and qRT–PCR analysis (Fig. 4H) in A549 and H460 cells. Thus, apatinib might promote the uptake and reprogram the metabolism of glutamine, which reduces its antitumor activity in NSCLC cells.

To further explore the metabolic flux of glutamine, we detected the ^13^C-labeled fractions of the TCA metabolites since majority of the carbon of glutamine enters the TCA cycle through metabolizing into α-KG. As shown in Fig. S5, the relative concentration of ^13^C-labeled α-KG increased upon apatinib treatment while other TCA metabolites (succinate, fumarate, malate, oxaloacetate and citrate) remained steady. We considered that there might be some metabolic bypasses of α-KG which were activated or the conversion between glutamate and α-KG reached to dynamic balance. Based on these results, we speculated that the increase of asparagine and glutamate, rather than the direct acceleration of the TCA cycle, lead to the resistance of apatinib.

### Apatinib promoted the uptake and catabolism of glutamine via the AAR pathway

**Fig. 5:**
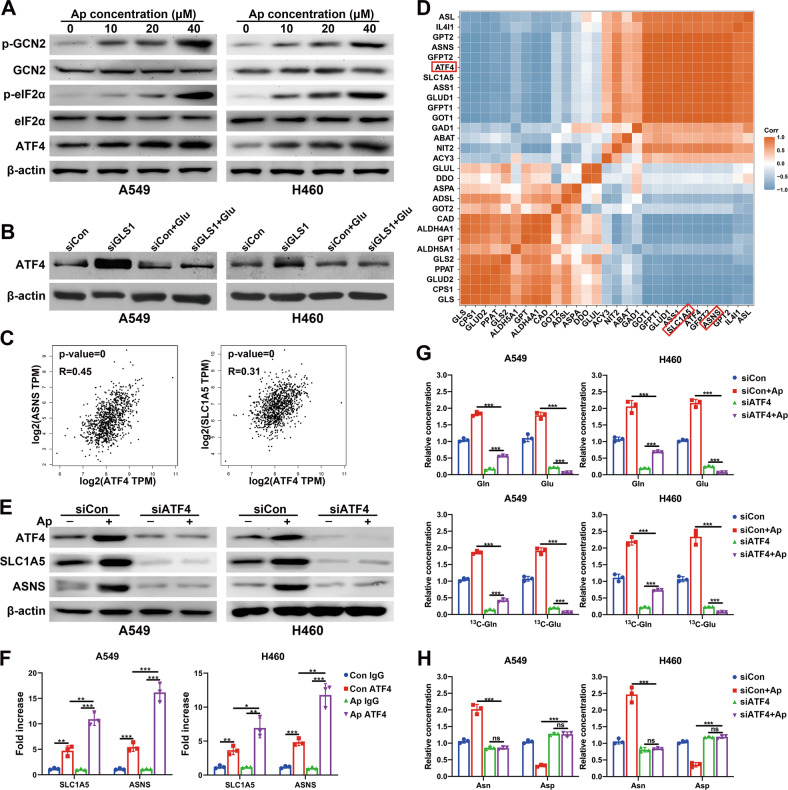
Apatinib promoted the uptake and catabolism of glutamine via the AAR pathway. **A** Protein expression of GCN2/eIF2α/ATF4 pathway genes in A459 and H460 cells incubated with increasing concentrations of apatinib (48 h) was detected by WB. **B** A549 and H460 cells were treated with or without siGLS1 or exogenous glutamate, and the protein levels of ATF4 were detected by WB. **C** Scatter plots present the correlations among ASNS (left), SLC1A5 (right), and ATF4 expression in NSCLC patients. **D** Pearson’s coefficient in the analysis of the RNA-seq data. **E** Corresponding cells were treated with or without siATF4 or apatinib, and the protein levels of ATF4, SLC1A5 and ASNS were detected by WB. **F** ChIP assay validated the binding capacity of ATF4 to the SLC1A5 and ASNS promoters in A459/H460 cells incubated with apatinib (20 μM, 48 h) or negative control. **G** LC–MS analysis of the relative intracellular contents of glutamine and glutamate in the control or siATF4 A459/H460 cells incubated with negative control or apatinib (20 μM, 48 h). **H** LC–MS analysis of the relative intracellular contents of asparagine and aspartate in control or siATF4 A459/H460 cells incubated with negatve control or apatinib (20 μM, 48 h). Data are presented as mean ± SEM from three independent experiments.

The AAR pathway, namely, the amino acid (starvation) response pathway, which is mainly referred to as the GCN2/eIF2α/ATF4 pathway, has been described to be activated under nutritional stress caused by amino acid deprivation [18]. In our study, we found that the protein expression levels of p-GCN2, p-eIF2α and ATF4 increased with apatinib treatment (Fig. 5A), which suggested the activation of the AAR pathway. Since eIF2α-ATF4 axis is an important metabolic stress pathway which can be activated by any of the four eIF2α kinase: GCN2, PERK, PKR, and HRI. To determine whether the increase of ATF4 was regulated by GCN2 under the treatment of apatinib, we knocked down GCN2 in both cell lines and found that the silence of GCN2 abolished the p-eIF2α and ATF4 induction upon apatinib treatment (Fig. S6A). GLS1 inhibition in triple-negative breast cancer (TNBC) cell lines has been reported to lead to the accumulation of ATF4 protein [19]. We further studied the relationship between GLS1 and ATF4 by knocking down GLS1 in both cell lines (Fig. S10C). The results showed that silence GLS1 would increase the protein level of ATF4. However, exogenous addition of glutamate reversed the upregulation of ATF4 (Fig. 5B).

ATF4 has been verified as a critical regulator of nutrient metabolism in many kinds of cancers, and its target genes include SLC1A5 and ASNS [20]. However, the relevance between them in lung cancer remains unclear. Therefore, the correlation between ATF4 and SLC1A5 in NSCLC patients were investigated through the GEPIA website (http://gepia.cancer-pku.cn) [21], and the results indicated that the mRNA expression level of ATF4 and SLC1A5 was positively correlated (*p* value < 0.0001, *R* = 0.45), as was the expression of ATF4 and ASNS (*p* value < 0.0001, *R* = 0.31) (Fig. 5C). To better define the amino acid metabolism response pathways, a gene-gene Pearson correlation analysis was performed using our RNA-seq data. As demonstrated in Fig. 5D, there was a strong positive correlation between the mRNA expression level of ATF4 and ASNS (Pearson’s *r* = 1), as well as SLC1A5 (Pearson’s *r* = 1). Furthermore, we investigated whether apatinib increased the expression of SLC1A5 and ASNS by activating ATF4. As shown in Fig. 5E, apatinib did not upregulate the expression of SLC1A5 or ASNS when ATF4 was silenced by siRNA.

Since ATF4 is a transcription factor, we speculated that apatinib treatment activated ATF4, which then activate target gene transcription through binding to their promotors. The binding sites of ATF4 on target gene promoters were analyzed with the online database JASPAR [22], multiple ATF4 binding sites were found on the SLC1A5 and ASNS promoters. Furthermore, ChIP assays confirmed the binding of ATF4 with the SLC1A5 and ASNS promoters. Upon apatinib treatment, the interactions between ATF4 and the SLC1A5 promoter and ASNS promoter were significantly increased (Fig. 5F).

Moreover, after ATF4 silencing, apatinib treatment led to a reduction in the intracellular glutamate concentration (Fig. 5G) but no significant changes in the contents of asparagine or aspartate (Fig. 5H). Glutamine accumulated since it could not metabolize into glutamate (Fig. 5G). The TCA intermediates pool was reduced upon apatinib treatment and ATF4 knockdown (Fig. S5). These results suggested that the combination of apatinib and ATF4 knockdown resulted in the thorough inhibition of glutamine metabolism in NSCLC.

### Combination of apatinib and ATF4 silencing contributed to cancer inhibition

**Fig. 6:**
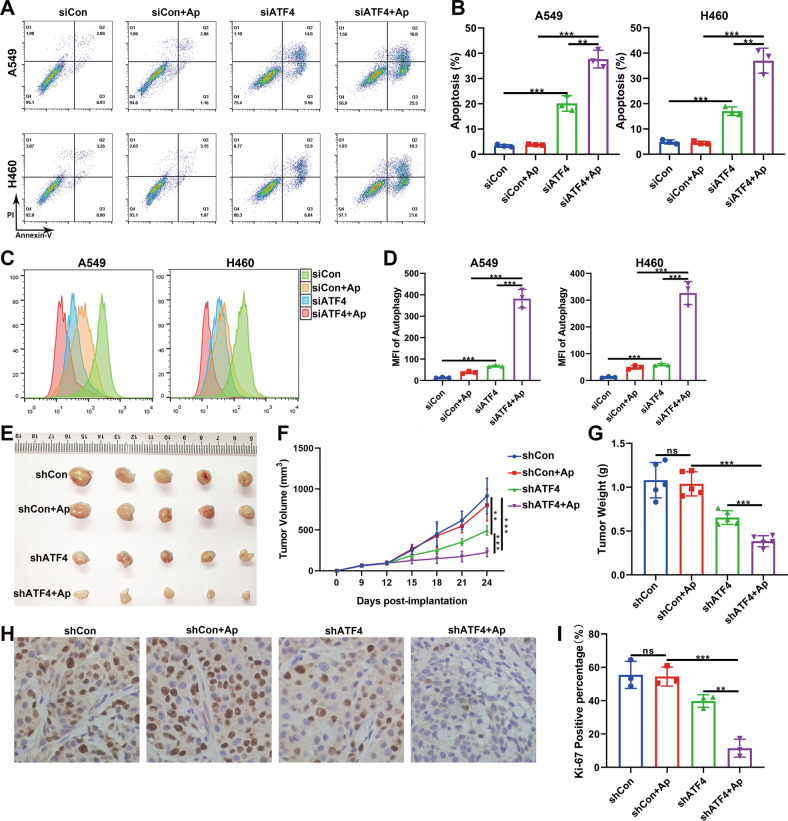
Combination of apatinib and ATF4 silencing contributed to cancer inhibition in vitro and in vivo. **A**, **B** A549 and H460 cells were treated with or without siATF4 or apatinib, and flow cytometry was performed to detect apoptotic cells. **C**, **D** A549 and H460 cells were treated with or without siATF4 or apatinib, and a Cyto-ID detection assay was performed to evaluate the autophagy level. **E** Mouse xenograft tumors were established using shCon-H460 cells or shATF4-H460 cells. After treatment with apatinib or saline, the xenograft tumors were harvested and the images were photographed. **F** Tumor volumes were scaled every 3 days, and tumor growth curves were drawn. **G** Weights of the xenograft tumors. **H**, **I** Representative IHC images and analysis of positive percentage of Ki-67 expression in xenograft tumors. Data are presented as mean ± SEM from three independent experiments.

Since the apatinib treatment and ATF4 knockdown led to the cutoff of glutamine metabolism, we speculated that cancer cells might have difficulty surviving in the absence of nutrition. Hence, we measured cell apoptosis by flow cytometry, and the results showed that the combination of ATF4 silencing and apatinib treatment led to significant apoptosis in NSCLC cells (Fig. 6A, B). The exogenous addition of glutamate and α-KG could partly reverse cell apoptosis (Fig. S7), while the addition of asparagine couldn’t. It might due to the unchanged concentration of asparagine in ATF4 silence and apatinib treatment group compared with the control group. These results demonstrated that the combination of ATF4 knockdown and apatinib treatment led to the depletion of glutamate and α-KG, which might be the leading cause of cell apoptosis. We have proven that apatinib can induce autophagy in NSCLC cells. However, whether autophagy is a protective mechanism or an inhibitory mechanism remains controversial. Here, our experiments showed that when nutritional stress was strengthened by ATF4 silencing, the autophagy level was significantly augmented (Fig. 6C, D). These results indicated that in the early stage of an energy crisis, the cells maintained viability through moderate autophagy. However, with the aggravation of nutritional deficiency caused by ATF4 knockdown, severe autophagy was induced, which may promote apoptosis.

We further conducted in vivo experiments to verify these results. NC-H460 cells or shATF4-H460 cells were inoculated into the BALB/C nude mice. The knockdown efficiency in H460 cells and xenograft tumors were presented in Figs. S10A and 10B. Saline or apatinib was intragastrically administered for 14 days. We found that although apatinib alone did not significantly inhibit the growth of transplanted tumors, the combination of shATF4 and apatinib suppressed tumor growth (Fig. 6E–G). As a proliferation marker, Ki-67 expression was found to be significantly reduced in the combination group (Fig. 6H, I). To further prove that the block of the AAR pathway in combination with apatinib treatment will increase the cancer inhibition, the GCN2 knockdown in NSCLC cells was also used and the results showed that apoptosis rate was significantly increased in vitro and the tumor growth was slowed in vivo upon combined treatment (Fig. S6B–E).

## Discussion

As an antiangiogenic drug, apatinib was also reported to have an antiproliferative effect on tumor cells. It simultaneously induced autophagy and apoptosis in thyroid cancer and osteosarcoma, in which autophagy was considered a protective mechanism, since inhibition of autophagy could promote apoptosis [12, 13, 23]. Moreover, these studies have shown that apoptosis could be triggered by apatinib at concentrations well below the IC50. However, we found that only high apatinib concentrations (much higher than the concentration that inhibited cell proliferation) could induce apoptosis in NSCLC cells. This finding implied that there might be a protective mechanism that was activated by apatinib. Moreover, apatinib was found to cause G2/M cell cycle arrest in osteosarcoma, hepatic carcinoma, and colorectal cancer [12, 13, 24], although it showed no significant effect on the cell cycle distribution in NSCLC in our experiment.

Glutamine and glucose are the primary nutrient sources for cancer cells and important for the biosynthesis of nucleic acids and nonessential amino acids (NEAAs). Our study showed that apatinib disrupted the balance of glutamine metabolism by activating the AAR pathway in NSCLC cells. Consistently, apatinib was reported to disturb pyruvate, alanine, aspartate, and glutamate metabolism in HepG2 cells [25], but the underlying mechanism was not revealed. Chen L et al. demonstrated that apatinib could inhibit glycolysis in ovarian cancer cells [14]. In our experiment, apatinib showed no significant effect on glucose uptake or glycolysis in NSCLC cells (Fig. S8). GLS1 (kidney type) and GLS2 (liver type) are the two main glutaminases in the human body. Normal cells mainly use GLS2 to decompose glutamine, while tumor cells depend on GLS1 [26]. Therefore, CB-839, a GLS1 inhibitor that blocks glutamine metabolism to inhibit tumor growth has entered phase I and II clinical trials [27]. However, studies have found that when glutamine deficiency occurs or when tumor cells are resistant to GLS1 inhibitors, cells rely on asparagine to escape death [28]. This finding is consistent with our experimental results that apatinib could inhibit GLS1 while promoting the production of asparagine by upregulating ATF4/ASNS. These results suggested that the inhibition of asparagine combined with apatinib may achieve synergistic antitumor effects.

Aspartate, one of the essential amino acids, has been found to be important for tumor proliferation. It has been reported that metformin inhibits tumor growth by consuming aspartate [29]. In an anoxic environment, the proliferation capacity of tumor cells was positively correlated with the aspartate content [30]. Arginine starvation induces ASNS expression, thus leading to aspartate depletion and promoting cell death, while aspartate supplementation or ASNS downregulation could protect arginine-deficient cells [31]. In human melanoma cells, when the synthesis of aspartate is inhibited, the GCN2/ATF4 pathway is activated to increase glutamine uptake by upregulating SLC1A5 [32]. In our study, apatinib reduced aspartate concentrations in NSCLC cells. Moreover, exogenous aspartate supplementation could reverse apatinib-induced autophagy (Fig. S9), which suggested that a decrease in aspartate was the main reason why apatinib caused autophagy.

As a key factor of the AAR pathway, ATF4 is overexpressed in a variety of solid tumors, suggesting that it plays an important role in tumor progression [20]. ATF4 can be activated when there is a lack of a variety of amino acids, thus making it the core regulator of amino acid metabolism. ATF4 can regulate a variety of amino acid transporters to maintain cell energy metabolism, such as upregulated leucine transporter SLC7A5, glutamine transporter SLC1A5, and cysteine-glutamate reverse transporter SLC7A11 [33]. Interestingly, these transporters are upregulated in cancers arising from different origins and under the influence of various oncogenic drivers. In triple-negative breast cancer, lower expression of SLC1A5 was correlated with better survival in both patients. Knockout SLC1A5 in mouse models also extend the survival time of mouse [34]. Studies have found that the mRNA level of ATF4 is positively correlated with the resistance of tumor cells to cisplatin. Recently, it has also been reported that the knockout of ATF4 can reduce the resistance of gastric cancer cells to cisplatin or increase the sensitivity of BRAF-mutated melanoma to vemurafenib [35, 36]. These studies indicated that ATF4 plays a pivotal role in cell resistance to chemotherapy drugs, which may be related to its function of promoting the production of metabolites.

ATF4 was reported to regulate a variety of autophagy-related genes. Its promoting or inhibiting effects on autophagy vary according to specific regulatory processes. It has been reported that after bortezomib treatment, ATF4 protects cells from bortezomib-induced apoptosis by promoting autophagy [37]. In another study, ATF4 knockout led to the inhibition of ASNS and a decrease in asparagine, thus leading to an increase in autophagy levels [38]. ATF4 has also been found to mediate the therapeutic resistance of temozolomide to glioma by upregulating SLC7A11 to absorb more cystine, promote the synthesis of glutathione, and neutralize reactive oxygen species. After ATF4 knockout, both autophagy and apoptosis of cells were increased, which removed the therapeutic resistance of temozolomide [39]. In our experiment, ATF4 knockdown inhibited the activation effect of apatinib on glutamine metabolism and a significant decrease in intracellular glutamate led to an increase in autophagy levels. When glutamine metabolism, a crucial energy source, was inhibited, cells could not obtain enough energy through autophagy. Excessive autophagy would eventually induce the activation of apoptosis.

**Fig. 7:**
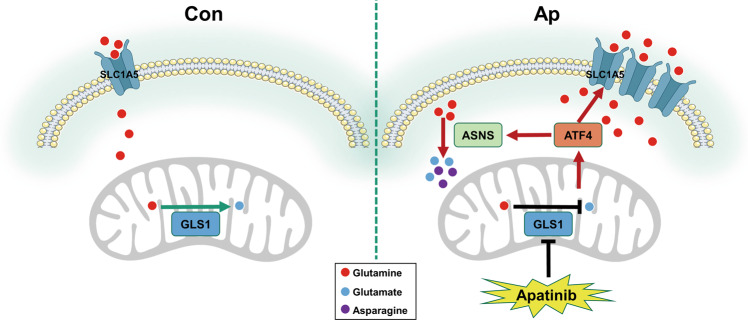
Mechanistic diagram of apatinib in NSCLC cells. Apatinib inhibits GLS1, which leads to the activation of ATF4. ATF4 upregulates SLC1A5 and ASNS by binding to their promoters. The increase in SLC1A5 contributes to the overabsorption of glutamine. The upregulation of ASNS promotes the reaction of glutamine and aspartate to generate glutamate and asparagine. Thus, apatinib enhances the uptake and catabolism of glutamine in NSCLC cells.

Taken together, we revealed that direct stimulation of the AAR pathway by apatinib in NSCLC promoted the uptake and reprogram the metabolism of glutamine and induced autophagy through the reduction of aspartate (Fig. 7). Furthermore, blocking the AAR pathway could stimulate cell apoptosis and improve the antitumor efficiency of apatinib both in vitro and in vivo, which provides a new strategy for combination therapy. In addition, our research highlights a novel perspective on the correlation between antiangiogenic therapy and tumor metabolism reprogramming, which needs to be further explored.

## Materials and methods

### Cell culture

Human NSCLC cell lines A549 and H460 were originally purchased from the cell bank of Chinese Academy of Sciences (Shanghai, China). Both cells were maintained in RPMI 1640 medium (containing 300 mg/L of glutamine) along with 10% fetal bovine serum. All cell lines were regularly tested for contamination with mycoplasma or other pathogens and authenticated using short tandem repeat (STR) profiling. For the ^13^C-labeled-glutamine flux experiment, we added the ^13^C-labeled-glutamine at the concentration of 300 mg/L into the glutamine-free RPMI medium. Two type of antibiotics (100 U/ml of penicillin and 100 mg/ml of streptomycin) were also supplemented to prevent contamination. The cells were maintained at 37 °C in a 5% CO_2_ atmosphere.

### Cell viability assay

3 × 10^3^ cells were added into 96-well cell culture plates. The cells would be cultured at 37 °C and 5% CO_2_ until attachment. Each well would be washed three time with medium to remove loosely attached and dead cells before the experiment. Then 100 μl medium containing DMSO, 5, 10, 20, and 40 μM apatinib were added into each well. Then 10 μl CCK-8 (Beyotime Biotechnology, Shanghai, China) were added into each well and mixed with the medium to incubated for 1 h. For colorimetric detection, the absorbance results were detected at 450 nm and the IC50 values were analyzed using Graphpad prism8.

### Colony formation assay

A total of 500 human NSCLC A549 and H460 cells were cultured in six-well plates at 37 °C, 5% CO_2_ overnight. After the cells were attached to the plate, the medium was discarded. Each well was washed three times before the medium with 0.1% DMSO or various concentration of apatinib were added into each well. The cells were changed into 4 ml fresh medium and cultured for 7 d after 48 h treatment. Then cells were counted after washed and stained with crystal violet (0.05%).

### EdU detection

A total of 2.5 × 10^4^ cells were cultured with apatinib (20 μM) for 48 h. EdU detection assay was carried out following the standard protocol of the Cell-Light EdU Apollo643 In Vitro Kit (RiboBio, Guangzhou, China). The A549 and H460 cells were incubated with pre-warmed medium containing EdU (50 μM). The cells were then incubated for 2 h, followed by aspirating culture medium and rinsing cells in PBS. After stopping the reaction, cells were fixed in 4% paraformaldehyde. The paraformaldehyde was then removed by washing. Then, the cells were permeated with 0.5% Triton X-100. The permeation solution was then removed by centrifuging the plate and rinsing cells in PBS. 100 μl Apollo was added into the cells and cultured for 30 min. Finally, the cells were cultured with Hoechst 33342 for 30 min to stain the nuclear. The counting of the positive cells was carried out using the CellProfiler software.

### Flow cytometric measurement of apoptosis and cell cycle

A total of 2 × 10^5^ A549 and H460 cells were added into 6-well plates and cultured at 37 °C, 5% CO_2_. At 70% confluence, apatinib was added into the well for 48 h, cell apoptosis was detected using Annexin V-FITC/PI apoptosis kit (BD Bioscience, Franklin Lake, NJ, USA) and cell cycle was determined after incubated with RNARase (5 mg/ml) and PI (1 mg/ml). After the treatment, all the A549 and H460 cells were collected and then analyzed by the FACS-Calibur flow cytometer (BD Bioscience, Franklin Lake, NJ, USA).

### Immunohistochemical (IHC) analysis

For IHC, the embedded xenografted tissues were cut into slides and incubate in blocking buffer. The slides then first incubated with primary body and then wash and incubated with HRP-labeled secondary antibody for 1 h. Next, the slides were counterstained with hematoxylin to visualize nuclei and photographed using microscope. The percentage of positive stained cells were calculated using CellProfiler.

### Cell autophagy analysis

The autophagy of cells was detected by following the standard protocol of Cyto-ID Autophagy Detection Kit (Enzo Life Sciences, Farmingdale, NY, USA). Briefly, 2 × 10^5^ cells were seeded into six-well plates and cultured at 37 °C, 5% CO_2_ until attachment. After treated with apatinib, the cells were incubated with staining agent for 30 min. The LC3II-positive punctate pattern was observed under confocal microscope (Carl Zeiss Laser Confocal Microscope, Oberkochen, German). The autophagic flux were assessed by FACS-Calibur flow cytometer.

For transmission electron microscopy (TEM) detection, the apatinib-treated A549 and H460 cells were placed in 2.5% glutaraldehyde and fixed for 1 h and then post-fixed in 1% osmium tetroxide. Fixed cells were embedded in agar and dehydrated in an ascending series of alcohols, and then embedded in epoxy resin. The sections were cut and placed on copper grids after stained with aqueous uranyl acetate and lead citrate. The images of sections were then collected under a Philips CM120 TEM (Philips, Amsterdam, Netherlands). The number of autophagosomes or autolysosomes in the cytoplasm of cells were calculated using CellProfiler.

### Measurement of intracellular metabolites

The intracellular levels of glutamine and glutamate were analyzed by UHPLC–MS/MS using an UHPLC system equipped with an amide column (100 × 2.1 mm, 3.5 μm; Waters, Milford, USA). For ^13^C tracing experiments, 5 × 10^5^ cells were cultured with RPMI medium containing 300 mg/L [U5]-^13^C glutamine or 2000 mg/L [U6]-^13^C glucose (Cambridge isotope laboratories, Tewksbury, UK). The samples were prepared and detected accordingly to the instructions of the manufacturer as described by Ma J, et al. [40].

The levels of common amino acids were measured by EZ: fast GC/FID Protein Hydrolysate Amino Acid Kit (Thermo Fisher Scientific, Cheshire, UK) by gas chromatography. The amino acid analysis was carried out following standard procedure as previously described by Akgül R, et al. [41].

### Glucose uptake assay

5 × 10^5^ A549 or H460 cells per well were cultured and treated with apatinib. The cells were harvested and incubation in 0.1 mM 2-NBDG (2-NBDG Glucose Uptake Assay Kit; Abcam, Cambridge, UK) for 15 min. Then the supernatant was discharged and the cells were rinsed two times with PBS and then collected for flow cytometry.

### Extracellular acidification rate (ECAR) analysis

The glycolytic rate of NSCLC cells in control or apatinib-treatment group was determined with an XF24 Extracellular Flux Analyzer (Agilent Technologies, Santa Clara, California, USA). Briefly, after incubated with 20 μM apatinib for 48 h, 2 × 10^4^ A549 cells or 2.5 × 10^4^ H460 cells were added into XF plates under condition at 37 °C, 5% CO_2_ for 12 h to attachment. Then, the cells were washed twice using seahorse detection medium supplemented with 2 mM glutamine. After that, the detection medium (500 μl/well) were added into cell and the cells were placed in a CO_2_ free biochemical incubator at 37 °C for 1 h. After base-line calibration, the ECAR was determined by adding 56 μl of 100 mM glucose, 62 μl of 10 M oligomycin and 69 μl of 500 mM 2-deoxyglucose to the cells.

### RNA interference

siATF4, siGLS1, siGCN2, and siNC were purchased from RiboBio (Guangzhou, China). The sequences are listed in Supplementary Table 3. 3.5 × 10^5^ A549 or H460 cells were transfected with siRNA following the manufacturer’s instructions. Briefly, the day before transfection, cells were seeded into six-well plate to reach 30–50% confluent the next day. For each well to be transfected, 30 pmol siRNA and 5 μl Lipofectamine RNAiMAX (Invitrogen, Carlsbad, CA, USA) was diluted in 2 tubes containing 250 μl Opti-MEM (Gibco, Invitrogen, Carlsbad, CA, USA) respectively. After 5 min, the diluted siRNA and Lipofectamine RNAiMAX was mixed gently and incubated for 20 min. Then the siRNA- Lipofectamine RNAiMAX were added to the wells containing cells and 1 ml complete medium. The knockdown efficiency was examined by WB 48 h later.

### Establishment of stable ATF4/GCN2 knockdown H460 cells

The stable ATF4 or GCN2 knockdown cell lines were established as previously described [42]. Briefly, HEK293T cells were transfected with the ATF4 and GCN2 shRNAs, packaged plasmids psPAX2 and pMD2.G. The shRNA sequence was listed in Supplementary Table 3. Then the cell supernatants were collected and filtered using a 0.45 μm filter. Next, H460 cells were infected using the filtered lentiviral supernatants adding 10 μg/mL of polybrene (Sigma, Santa Clara, CA, USA). After 72 h, cells were cultured and screened using complete medium containing 2 μg/mL puromycin for 48 h. Then the knockdown efficiency was examined by WB.

### Real-time qPCR

2 × 10^5^ cells were seeded into six-well plates and cultured overnight. After treated with apatinib or DMSO, the cells were collected and then incubated with 1 ml TRIzol Reagent and 200 μl chloroform. The A549 or H460 cells were centrifuged and the supernatant were collected into a new RNA-free tube. The sample were added with equal isopropanol into the tube. After gentle inversion, the sample was frozen and then centrifuged. Subsequently, the sample was washed with precooled 75% ethanol, and then DEPC water was used to dilute RNA to final concentration of 1 ug/ul. For reverse transcription PCR, the procedures were performed following the standard protocol of the TaKaRa qPCR kit (Takara Bio Inc., Kusatsu, Shiga, Japan). The primers were listed in Supplementary Table 1. The relative mRNA expression levels were calculated as described before [43].

### RNA sequencing and bioinformation analysis

5 × 10^6^ A549 or H460 cells were seeded in the Petri dishes (10 cm diameter). After treated with apatinib, the total RNA was extracted. RNA sequencing and library construction were performed according to the Wuhan Huada Sequencing Company’s instructions (BGI, Shenzhen, China). Cutoff log2 (Fold Change) > 1 and *p* value < 0.05 were applied to determine the DEGs between apatinib treatment and control group. The raw data were uploaded to NCBI Bioproject (Accession code: PRJNA782197).

### Chip assay

5 × 10^6^ A549/H460 cells were seeded in the Petri dishes (10 cm diameter) and then scripted from the plate after treated with apatinib or DMSO. The cells were resuspended and then incubated with 1% formaldehyde for 10 min to ensure the DNA and protein cross-linked. The samples were sonicated 14 times for 20 s each at 25% setting to shear chromatin. Adding the Protein A/G beads into the chromatin and mixed at 4 °C for 1 h. Then, the DNA-protein complex was immunoprecipitated with ATF4 antibody or negative control rabbit IgG antibody. The precipitated chromatin was purified, de-crosslinked, and analyzed by qPCR. The primers were designed to analyze ATF4-binding sites near the promoter region (1000 bp upstream of each site) of the target genes. The sequences were listed in Supplementary Table 4.

### Western blot analysis

After treatment with apatinib or DMSO, 5 × 10^5^ A549 and H460 cells were washed in PBS and lysed with RIPA buffer containing 1 mM PMSF on ice for 30 min. Then, the lysed cells were collected and centrifuged (12,000 × *g* for 25 min) to separate the supernatant. After detecting the concentration, the protein was mixed up with loading buffer and denatured at 100 °C for 5 min. For the western blot, equal amount (50 μg) of protein extract was added and separated using the precast SDS–PAGE for electrophoresis and transferred to the polyvinylidene fluoride membranes. The membranes were blocked with 5% skim milk under vibration, then incubated with the specific primary antibodies and a secondary antibody. Antibodies used in this study were listed in Supplementary Table 2. In the final step, the bands detection was performed using the chemiluminescence detection system.

### Animal experiments

Human NSCLC H460 cells were stably transfected with lentiviral vectors encoding shATF4/shGCN2 or empty control lentiviral vectors. The number of transfected cells were quantitated with a Coulter counter before the H460 cells (5 × 10^6^ per mouse) were injected into the BALB/c mice (6-week-old, female). The animal experiments were conducted with the approval of the Institutional Animal Ethics Committee of Huazhong University of Science and Technology. The injection site is subcutaneous at the right flank of the mice and were randomly divided into four groups. Five mice were used in each experimental group. Apatinib (50 mg/kg, 100 µl/d) or equal PBS were administered to the corresponding group intragastrically. At two weeks following implantation (tumor size approximately 100 mm^3^), a tumor would begin to appear at the site of implantation. The size of tumor was measured every 3 days for two perpendicular tumor diameters. The volume of xenografted NSCLC H460 tumor was calculated based on the formula: volume = 1/2 × length × width^2^. All mice were euthanized on the 24th day.

### Statistical analysis

All results were present as the mean ± SEM from triplicate experiments. Two-tailed Student’s t test and one-way ANOVA were conducted to analyze all data. All data met the assumptions of the tests and statistical tests were justified as appropriate. A significant difference is indicated at *P < 0.05, **P < 0.01, ***P < 0.001.

## Supplementary information

Reproducibility checklist

Supplementary Tables

Supplementary Figures

Original Data File

## Data Availability

The data used in this study are available from Zhenyu Li or Gang Wu on reasonable request.
