# Supplementary material for: Activated amino acid response pathway generates apatinib resistance by reprograming glutamine metabolism in non-small-cell lung cancer
Source: Cell Death Dis. 2022 Jul 21;13(7):636. doi: 10.1038/s41419-022-05079-y (PMC9304404; doi:10.1038/s41419-022-05079-y)
Supplement: Supplementary file 2 — Supplementary Tables [file 41419_2022_5079_MOESM2_ESM.docx]

**Supplementary Table 1. The primer pairs for RT-PCR**

| **Gene** | **Sequence (5’-3’)** | | |
| --- | --- | --- | --- |
| ATF4 | F: | GGTTCTCCAGCGACAAGG |  |
|  | R: | TCTCCAACATCCAATCTGTCC |  |
| ASNS | F: | GGAAGACAGCCCCGATTTACT |  |
|  | R: | AGCACGAACTGTTGTAATGTCA |  |
| ASNS (for CHIP) | F: | CAGGGTGATGTGGCGGGCTGAGG |  |
|  | R: | TTAAACAGGCGCACTGAGACGCA |  |
| SLC1A5 | F: | CAACCTGGTGTCAGCAGCCTT |  |
|  | R: | GCACCGTCCATGTTGACGGTG |  |
| SLC1A5 (for CHIP) | F: | TGCTGTAGGAGGAAGCTTCTG |  |
|  | R: | CGGTTACCAGCCAGAGAAAG |  |
| GLS1 | F: | ACUAUCAUUAUCUGAAGGGTT |  |
|  | R: | CCTTTGATCACCACCTTCTCTTCGA |  |
| GLUT1 | F: | GGCCAAGAGTGTGCTAAAGAA |  |
|  | R: | ACAGCGTTGATGCCAGACAG |  |
| GLUT4 | F: | TGGGCGGCATGATTTCCTC |  |
|  | R: | GCCAGGACATTGTTGACCAG |  |
| HK2 | F: | GAGCCACCACTCACCCTACT |  |
|  | R: | CCAGGCATTCGGCAATGTG |  |
| PFKL | F: | GCTGGGCGGCACTATCATT |  |
|  | R: | TCAGGTGCGAGTAGGTCCG |  |
| PKM2 | F: | ATGTCGAAGCCCCATAGTGAA |  |
|  | R: | TGGGTGGTGAATCAATGTCCA |  |
| β-actin | F: | CAACCGCGAGAAGATGACC |  |
|  | R: | ATCACGATGCCAGTGGTACG |  |

**Supplementary Table 2. The primary antibodies for WB**

| **Antibody** | **Cat No.** | **Manufacturer** |
| --- | --- | --- |
| LC3B | ab192890 | Abcam |
| GLS1 | ab156876 | Abcam |
| SLC1A5 | 8057 | CST |
| GCN2 | 65981 | CST |
| p-GCN2 | 94668 | CST |
| eIF2α | 5324 | CST |
| p-eIF2α | 3398 | CST |
| ASNS | 92479 | CST |
| ATF4 | 10835-1-AP | Proteintech |
| caspase3 | AC030 | Beyotime Biotechnology |
| cleaved-caspase3 | 9654 | CST |
| β-Actin | sc-8432 | Santa Cruz |

**Supplementary Table 3. The siRNA and shRNA sequences used in this study**

| **Genes** | | **Sequence (5’-3’)** |
| --- | --- | --- |
| ATF4 (si) | CAGAUUGGAUGUUGGAGAA | |
| ATF4 (sh) | TGCTTACGTTGCCATGATC | |
| GLS1 (si) | GGGUCUGUUACCUAGCUUG | |
| GCN2 (si) | CCAUCUACACCAUCUAUGA | |
| GCN2 (sh) | TGGCTAAGCAGGAACGTTT | |

**Supplementary Table 4. The conserved ATF4 binding motifs on ASNS and SLC1A5**

| **Genes** | | **Binding motifs** |
| --- | --- | --- |
| ASNS | TCAAAGTAGTAC | |
| SLC1A5 | CATGGTGAAACC | |
