# Supplementary material for: Activated amino acid response pathway generates apatinib resistance by reprograming glutamine metabolism in non-small-cell lung cancer
Source: Cell Death Dis. 2022 Jul 21;13(7):636. doi: 10.1038/s41419-022-05079-y (PMC9304404; doi:10.1038/s41419-022-05079-y)
Supplement: Supplementary file 3 — Supplementary Figures [file 41419_2022_5079_MOESM3_ESM.docx]

**Supplementary Figures**


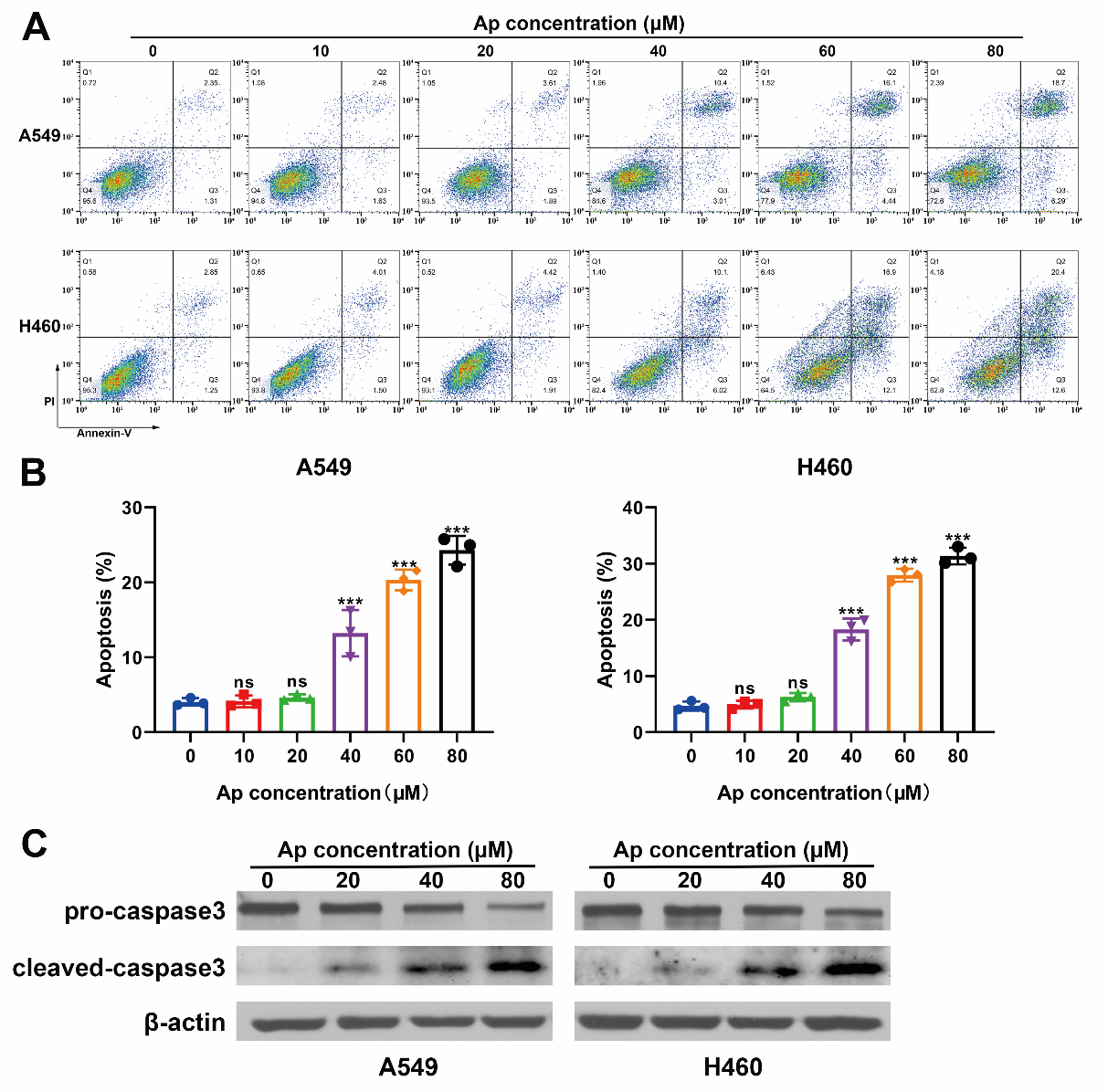


**Fig. S1** Low doses of apatinib did not induce apoptosis in NSCLC cells. (A) A549 and H460 cells were incubated with apatinib at different concentrations (0, 5, 10, 20, 40, 60 and 80 μM) for 48 h. Cell apoptosis was detected by flow cytometry. (B) Statistical analysis of the percentage of cell apoptosis. (C) The expression level of caspase3 in apatinib-treated (0, 20,40 and 80μM for 48 h) cells detected by WB. Data are presented as mean ± SEM from three independent experiments.


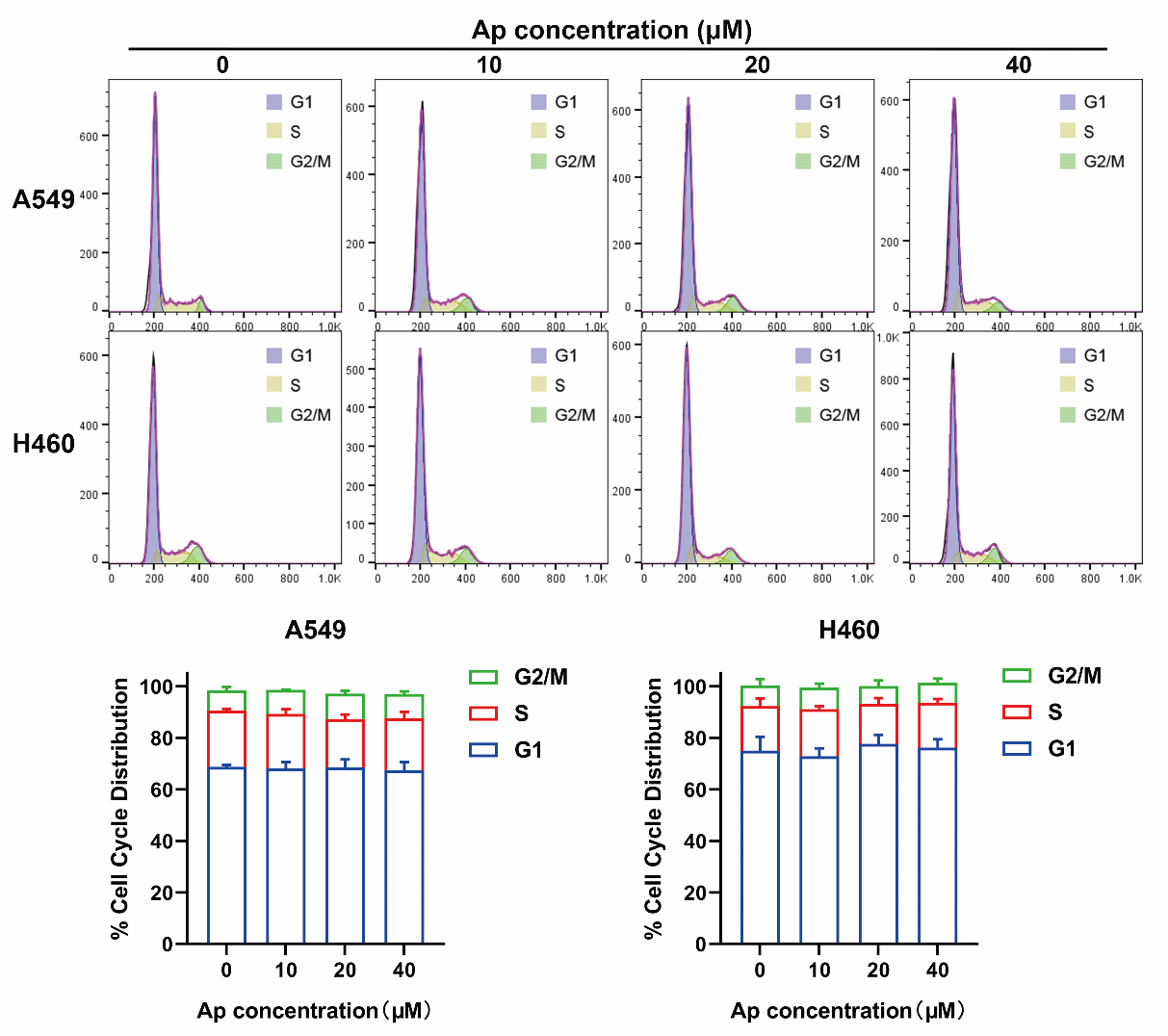


**Fig. S2 Apatinib did not induce cell cycle redistribution in NSCLC cells.** A549 and H460 cells were treated with apatinib at different concentrations (0, 5, 10, 20, 40 μM) for 48 h. Cell cycle distribution was analyzed by flow cytometry. Data are presented as mean ± SEM from three independent experiments.


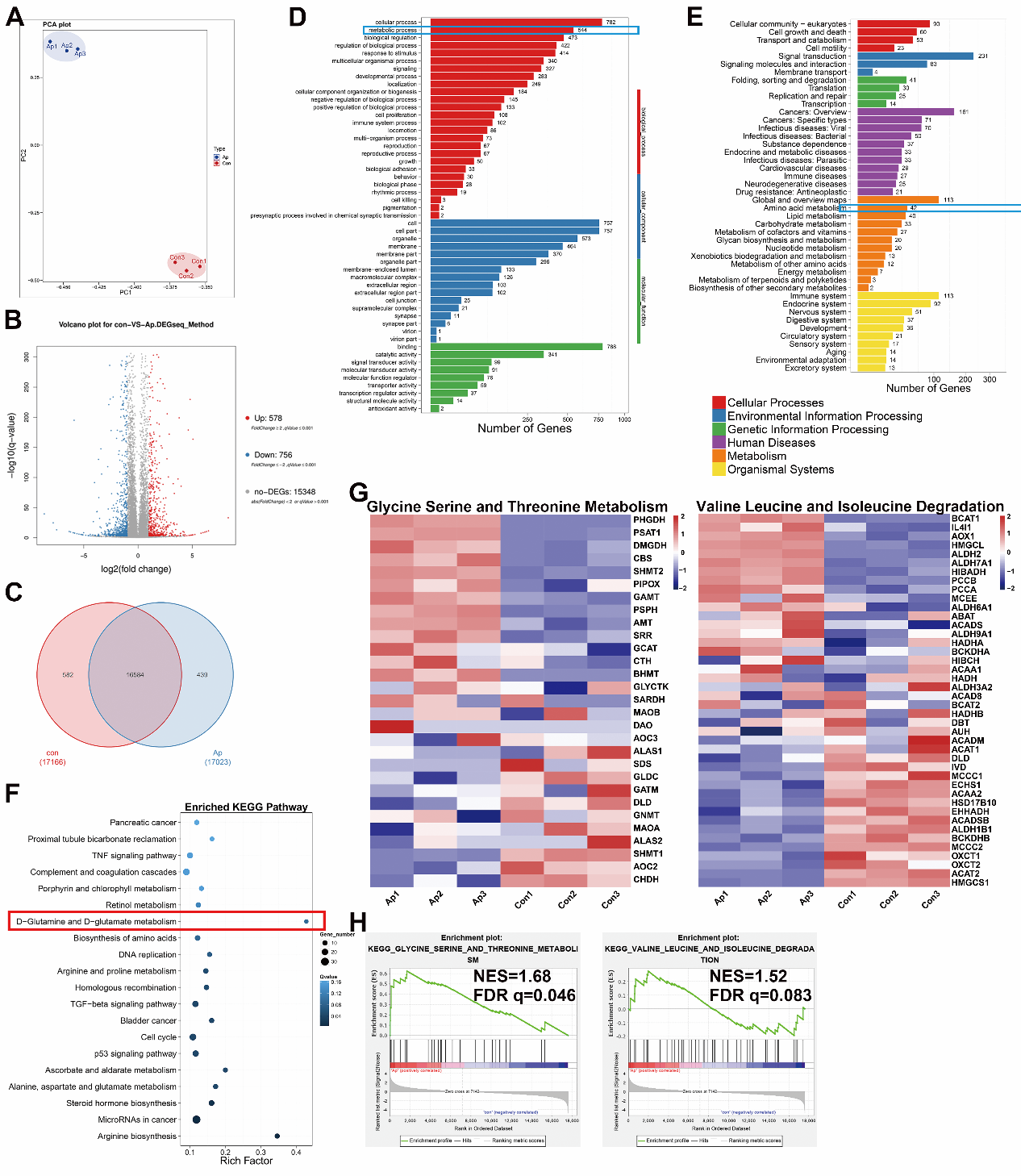


**Fig. S3 Apatinib activated amino acid metabolism in A549 cells.** (**A**) Principal component analysis (PCA) scatter plot of the differentially expressed genes (DEGs) of A459 cells with negative control or apatinib treatment (20 μM, 48 h). (**B**) Volcano plot of the DEGs. (**C**) Venn diagram of the overlapping DEGs. (**D**) GO analysis of the DEGs. (**E-F**) KEGG enrichment analysis and bar plot of the DEGs. (**G**) Left: Heatmap of relative mRNA expression of glycine, serine and threonine metabolism-related genes. Right: Heatmap of relative mRNA expression of valine, leucine and isoleucine metabolism-related genes. (**H**) Left: GSEA analysis of glycine, serine and threonine metabolism-related genes. Right: GSEA analysis of valine, leucine and isoleucine metabolism-related genes. NES, normalized enrichment score; FDRq, FDR statistical value. Data are presented as mean ± SEM from three independent experiments.


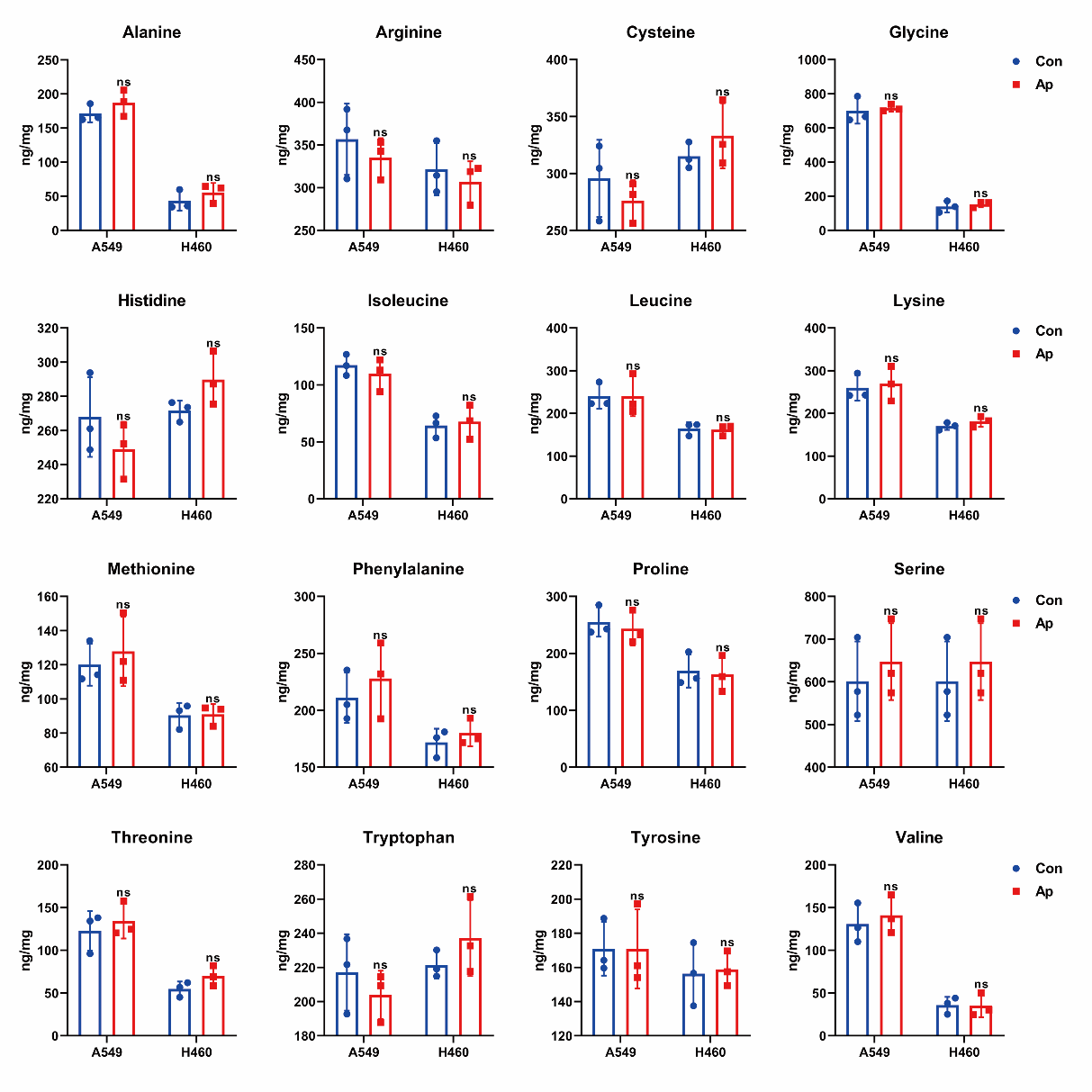


**Fig. S4 Apatinib did not change the concentrations of the other 16 common amino acids.** The intracellular concentrations of indicated amino acid in A459 and H460 cells incubated with negative control or apatinib (20 μM, 48 h) were detected by GC-FID. Data are presented as mean ± SEM from three independent experiments.


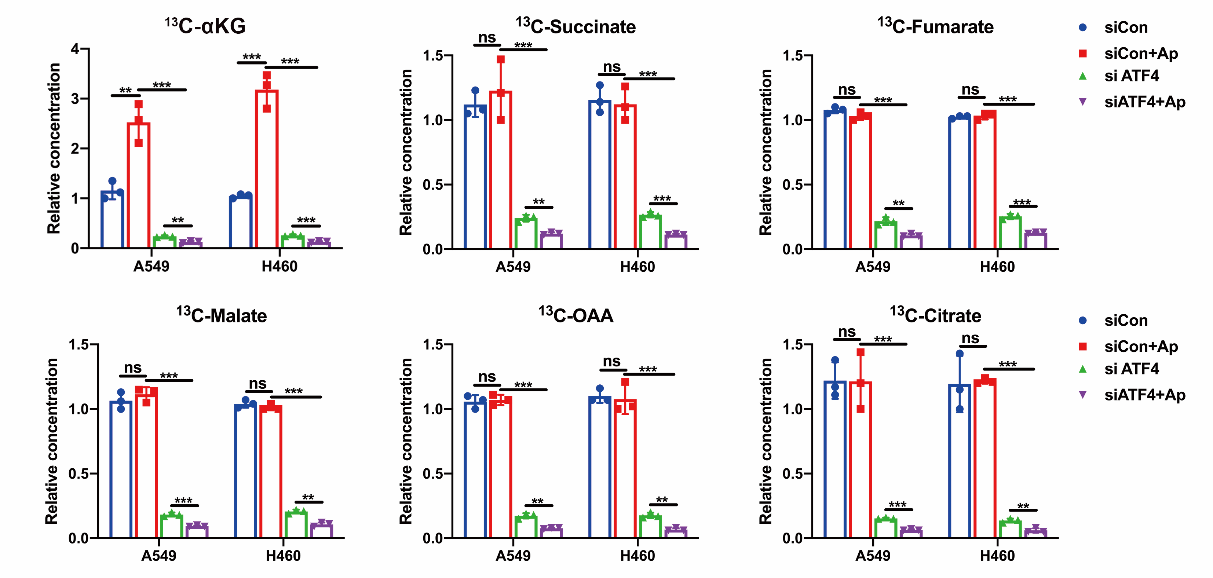


**Fig. S5 Apatinib treatment upregulated the concentrations of** **αKG while didn’t influence other TCA metabolites.** The intracellular concentrations of ^13^C-labeled-glutamine flux of the TCA metabolites (αKG, succinate, fumarate, malate, OAA and citrate) in A459 and H460 cells upon apatinib treatment (20 μM, 48 h) and ATF knockdown were detected by LC-MS. Data are presented as mean ± SEM from three independent experiments.


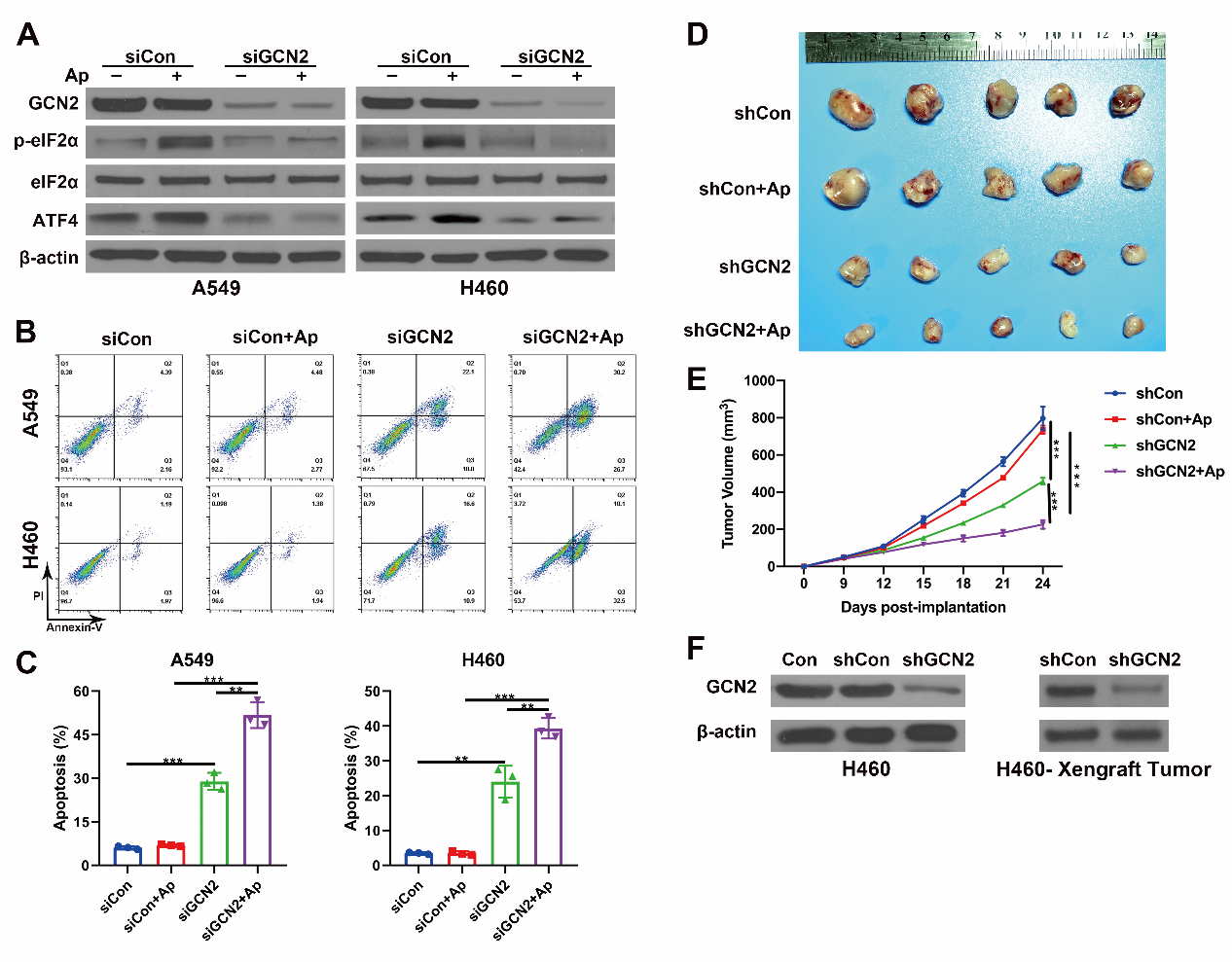


**Fig. S6 GCN2 knockdown abolished the p-eIF2α and ATF4 induction and promoted apoptosis synergistically with apatinib *in vitro* and *in vivo*.** (**A**) The negative control or GCN2 knockdown cells were treated with or without apatinib (20 μM, 48 h). The protein expressions of GCN2/eIF2α/ATF4 pathway genes were detected by WB. (**B-C**) Flow cytometry was performed to detect apoptotic cells. (**D**) Mouse xenograft tumors were established using shCon-H460 cells or shGCN2-H460 cells. After treatment with apatinib or saline, the xenograft tumors were harvested and the images were photographed. (**E**) Tumor volumes were scaled every 3 days, and tumor growth curves were drawn. (**F**) The knockdown efficiency of GCN2 was evaluated by WB in H460 cells and H460-derived xenograft tumors. Data are presented as mean ± SEM from three independent experiments.


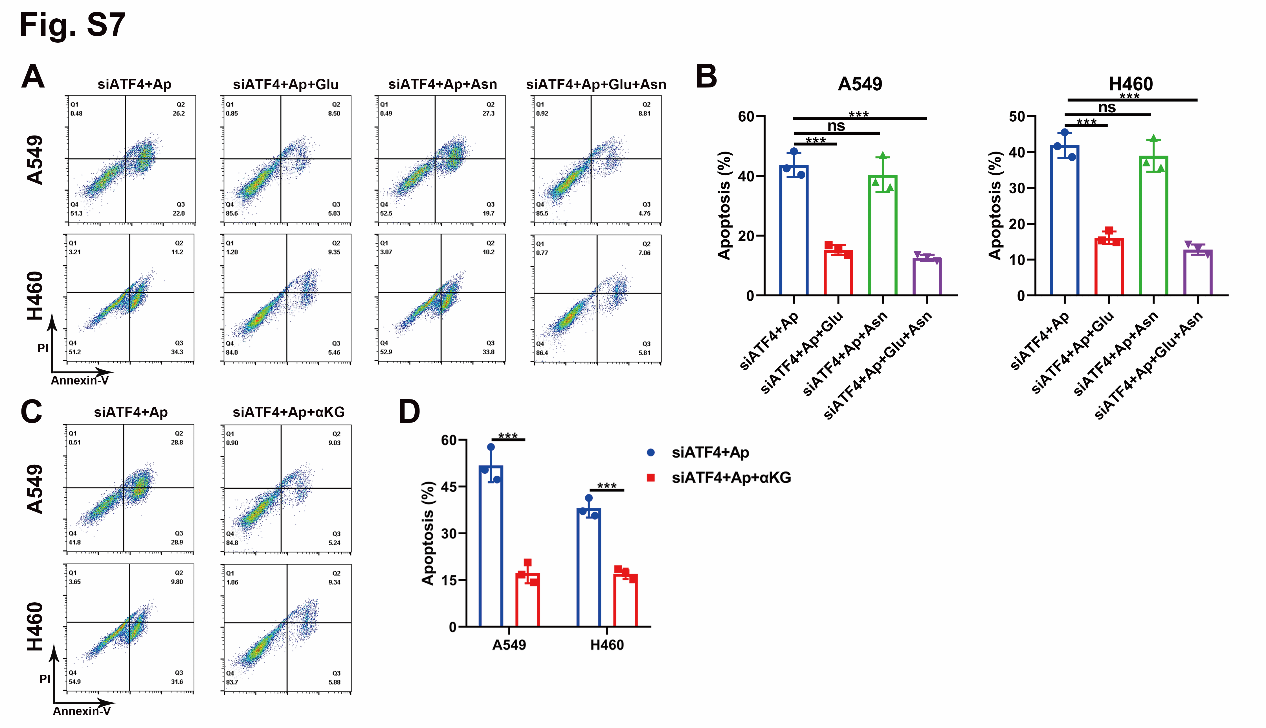


**Fig. S7 Exogenous glutamate and αKG supplementation reversed apatinib and ATF4 knockdown induced apoptosis while asparagine didn’t.** (**A-B**) Upon ATF4 knockdown, A549 and H460 cells were incubated with 20 μM apatinib, 20 mM glutamate and 1 mM asparagine for 48 h. Flow cytometry was performed to detect cell apoptosis. (**C-D**) Upon ATF4 knockdown, A549 and H460 cells were incubated with 20 μM apatinib and 15mM α-KG for 48 h. Flow cytometry was performed to detect apoptosis. Data are presented as mean ± SEM from three independent experiments.


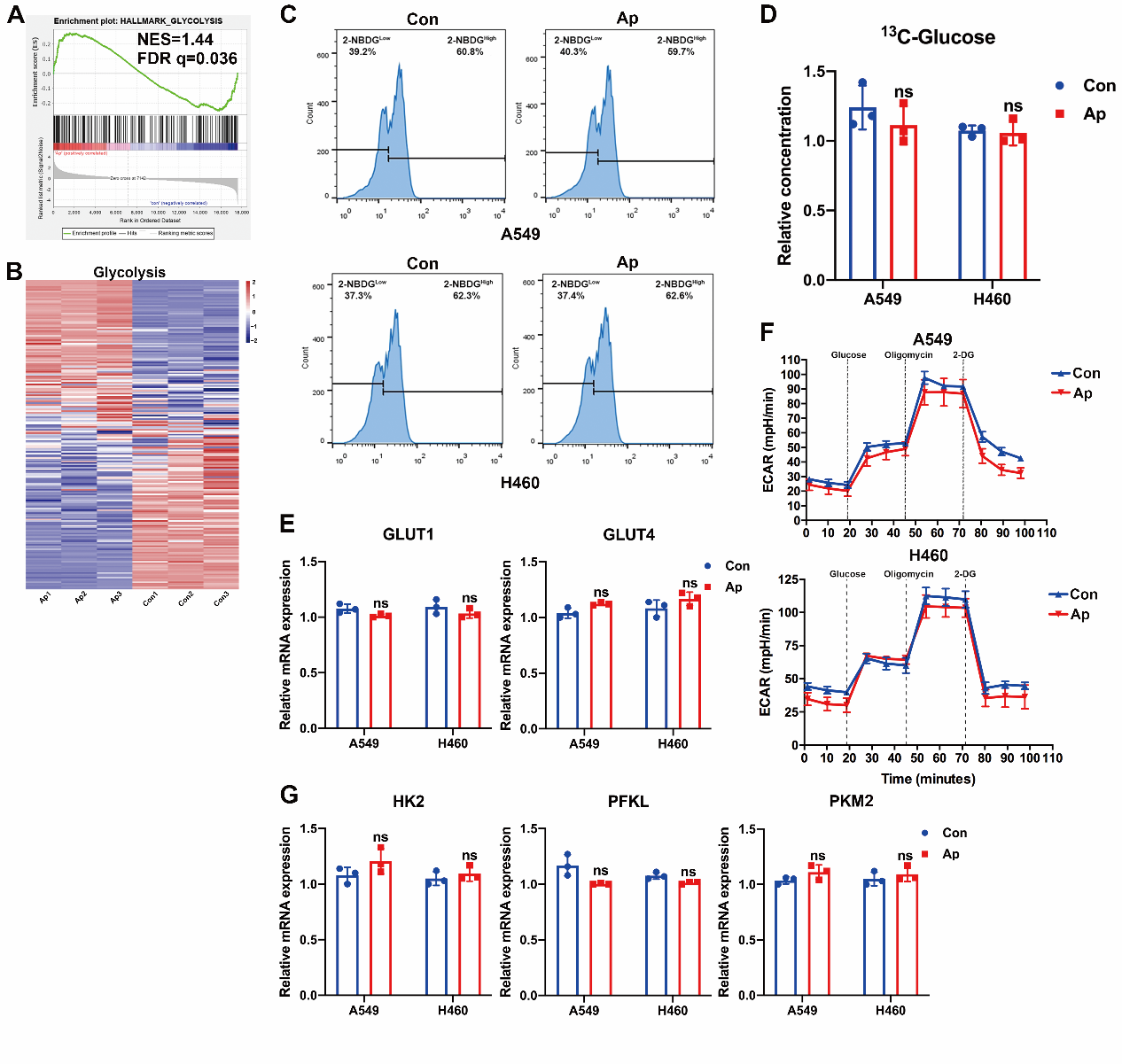


**Fig. S8 Apatinib did not affect glycolysis in NSCLC cells.** (**A**) GSEA of glycolysis related genes. NES, normalized enrichment score; FDRq, FDR statistical value. (**B**) Heatmap of the relative mRNA expression of glycolysis-related genes. (**C**) Cells were stained with 2-NBDG, and the amount of fluorescence was measured by flow cytometry to estimate the uptake of glucose. (**D**) LC–MS analysis of relative intracellular concentration of ^13^C-glucose in A459 and H460 cells treated with or without apatinib (20 μM, 48 h). (**E**) qRT-PCR analysis of the relative mRNA expression of the glucose transpoters (GLUT1 and GLUT4) in A459 and H460 cells treated with or without apatinib (20 μM, 48 h). (**F**) ECAR levels, which represent the glycolytic rate, were determined by a seahorse glycolysis stress assay. (**G**) qRT-PCR analysis of the relative mRNA expression of the key enzymes (HK2, PFKL and PKM2) of glycolysis in A459 and H460 cells treated with or without apatinib (20 μM, 48 h). Data are presented as mean ± SEM from three independent experiments.


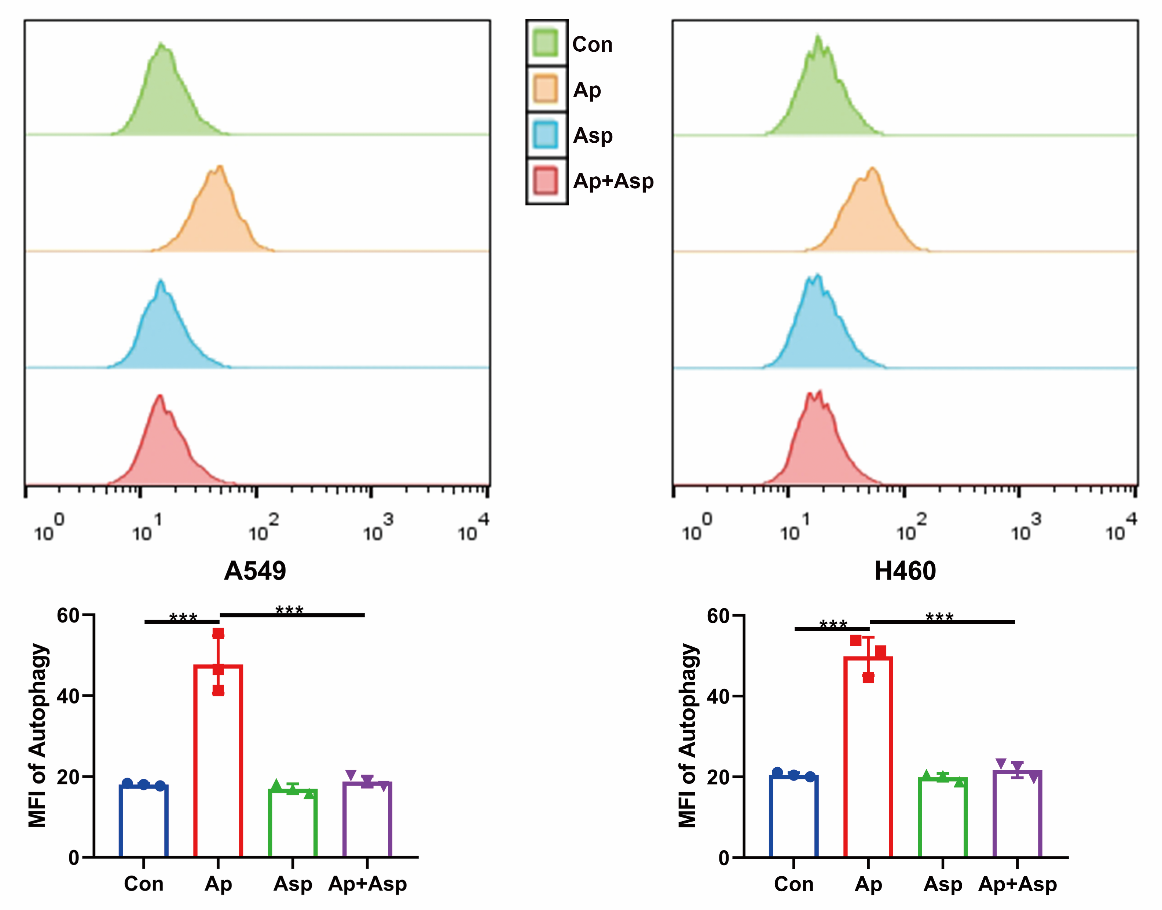


**Fig. S9 Exogenous aspartate supplementation reversed apatinib-induced autophagy.** Cyto-ID detection assay measured the autophagy levels of A549 and H460 cells with or without exogenous asparate supplementation under the treatment with 20 μM apatinib for 48 h. Data are presented as mean ± SEM from three independent experiments.


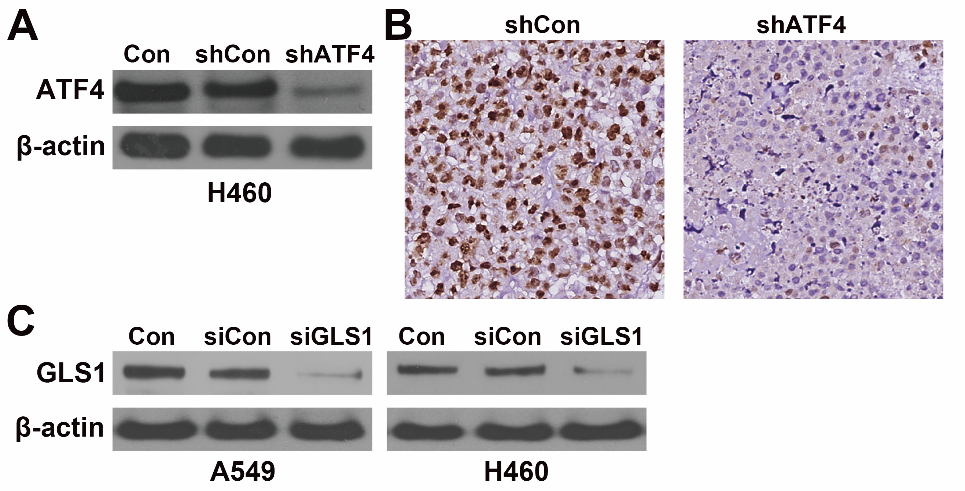


**Fig. S10** **Evaluation of the knockdown efficiency of ATF4 or GLS1.** (**A**) The knockdown efficiency of ATF4 was evaluated by WB in H460 cells. (**B**) The knockdown efficiency of ATF4 was presented by IHC in H460-derived xenograft tumors. (**C**) The knockdown efficiency of GLS1 was evaluated by WB in A549 and H460 cells. Data are presented as mean ± SEM from three independent experiments.
