# Supplementary figures and images for: Activated amino acid response pathway generates apatinib resistance by reprograming glutamine metabolism in non-small-cell lung cancer
Source: Cell Death Dis. 2022 Jul 21;13(7):636. doi: 10.1038/s41419-022-05079-y (PMC9304404; doi:10.1038/s41419-022-05079-y)

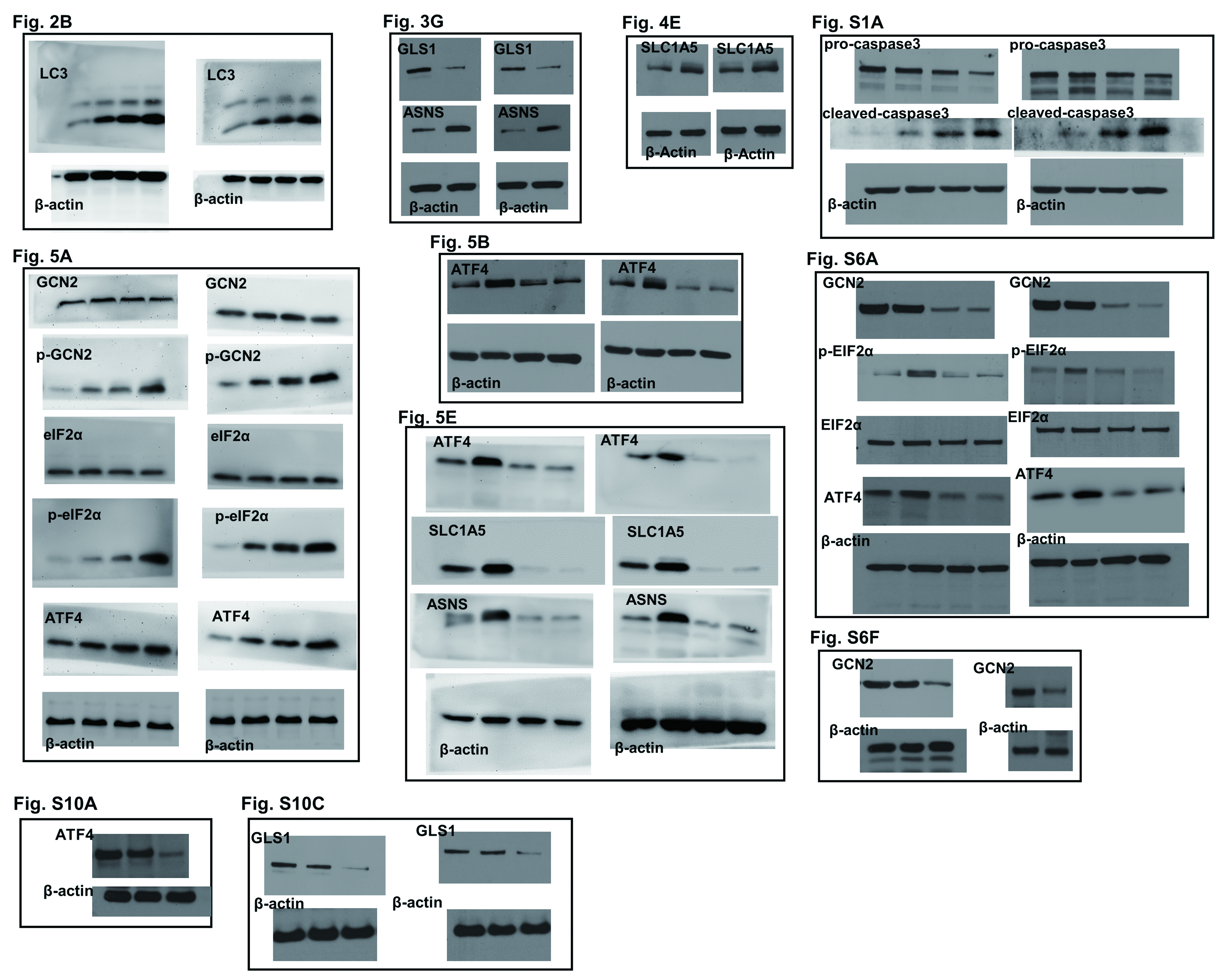

Supplement: Supplementary file 4 — Original Data File [file 41419_2022_5079_MOESM4_ESM.tif]
